# Shared-care survivorship program for testicular cancer patients: safe and feasible

**DOI:** 10.1016/j.esmoop.2022.100488

**Published:** 2022-05-13

**Authors:** H. Boer, S. Lubberts, S. Bunskoek, J. Nuver, J.D. Lefrandt, G. Steursma, W.J. Sluiter, S. Siesling, A.J. Berendsen, J.A. Gietema

**Affiliations:** 1 Department of Medical Oncology, University Medical Center Groningen, University of Groningen, Groningen, The Netherlands; 2 Department of Vascular Medicine, University Medical Center Groningen, University of Groningen, Groningen, The Netherlands; 3 Department of Endocrinology, University Medical Center Groningen, University of Groningen, Groningen, The Netherlands; 4 Department of Research and Development, Netherlands Comprehensive Cancer Organization, Utrecht, The Netherlands; 5 Department of Health Technology and Services Research, Technical Medicine Center, University of Twente, Enschede, The Netherlands; 6 Department of General Practice, University Medical Center Groningen, University of Groningen, Groningen, The Netherlands

**Keywords:** testicular cancer, cancer survivorship

## Abstract

**Background:**

Testicular cancer survivors are at risk for cardiovascular disease, often preceded by early development of cardiovascular risk factors due to chemotherapeutic treatment. Therefore, close collaboration between oncologists and primary care physicians (PCPs) is needed during follow-up to monitor and manage cardiovascular risk factors. We designed a shared-care survivorship program, in which testicular cancer patients visit both their oncologist and their PCP. The objective of this study was to test the safety and feasibility of shared-care follow-up after treatment for metastatic testicular cancer.

**Patients and methods:**

The study was designed as an observational cohort study with a stopping rule to check for the safety of follow-up. Safety boundaries were defined for failures in the detection of signals indicating cancer recurrence. Secondary outcomes were the proportion of carried out cardiovascular risk assessments, psychosocial status and patient preferences measured with an evaluation questionnaire.

**Results:**

One hundred and sixty-two patients were enrolled (69% of eligible testicular cancer patients). Almost all (99%, *n* = 150) PCPs of the enrolled patients agreed to participate in the study. In total, 364 primary care visits took place. No failures occurred in the detection of relapsed testicular cancer. Four follow-up visits were considered as failures because of organizational issues, without activation of the stopping rule. Eventually, the safe boundary was crossed indicating that this shared-care model is a safe alternative for follow-up after testicular cancer. Patients were satisfied with the knowledge level of PCPs. PCPs were willing to further extend their role in follow-up care after cancer.

**Conclusions:**

Shared-care follow-up is safe and feasible in this patient population. Patients benefit from personalized care, partly close to their home. Within shared care, PCPs can have an important role in cardiovascular risk management and psychosocial survivorship issues.

## Highlights

• Shared-care follow-up by oncologists and PCPs is safe and feasible for testicular cancer patients.
• The participation of PCPs in survivorship care improves cardiovascular risk management.
• PCPs are willing to expand their role in survivorship care.
• Less visits to the oncologist did not increase anxiety in testicular cancer survivors.

## Introduction

The growing population of cancer survivors is in need of high-quality survivorship care: detection of potential cancer recurrence, regaining of general health and screening for psychosocial issues and potential late effects of treatment for which intervention strategies are available.^1^

Patients with metastatic testicular cancer have an excellent prognosis, due to the success of platinum-based combination chemotherapy.^2^ Testicular cancer survivors are subjected to an intense follow-up schedule for 10 years after chemotherapy, because curative treatment is still possible when relapses are detected early. During follow-up, a substantial number of long-term survivors develop late toxicity.^3^ An important late effect of platinum-based chemotherapy is cardiovascular morbidity. During the first years of follow-up, many testicular cancer survivors develop various cardiovascular risk factors, often clustered into the metabolic syndrome.^4^ Follow-up care is usually concluded after 10 years, although a substantial number of cardiac events are observed >10 years after treatment.^5^

This development of cardiovascular risk factors in survivors of testicular cancer calls for better collaboration during follow-up care between the oncologist and the primary care physician (PCP). We hypothesized that shared-care follow-up results in better survivorship care. The PCP may be better equipped for the management of cardiovascular risk factors and other co-morbid conditions. Patients may also benefit from the generalist’s view of PCPs: psychosocial support, long-term continuity of care—also after completion of the regular follow-up—and from provision of care closer to home.^6^ Shared-care follow-up is successfully applied, for example, for breast cancer and childhood cancer survivors, but this is not yet the case for testicular cancer.^7^, ^8^, ^9^ Therefore, we designed a survivorship care program for testicular cancer survivors to test the safety and feasibility of shared-care follow-up together with the PCP, and compared this with standard oncologist-only follow-up.

## Patients and methods

### Design and study population

**Figure 1:**
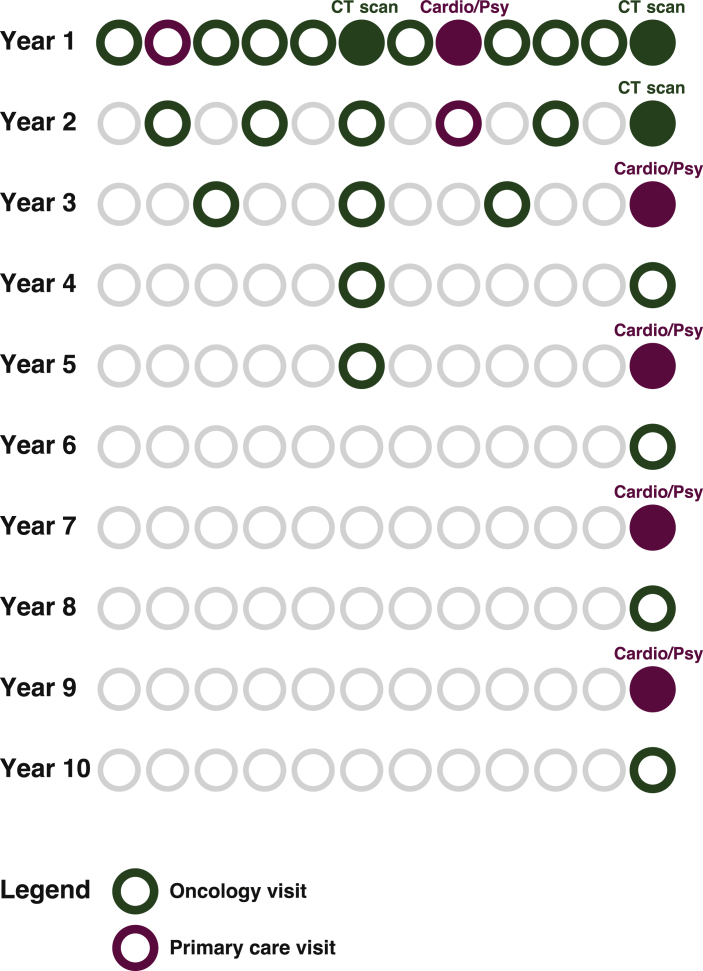
**Design of shared-care survivorship program.** Cardio, cardiovascular risk screening; CT, computed tomography; Psy, psychosocial questionnaires.

The study was designed as an observational cohort study (clinicaltrials.gov: NCT01783145). Patients were asked to participate in a shared-care survivorship program. Follow-up visits in the program included physical examination, measurement of tumor markers and computed tomography (CT) scans (routinely carried out at 6, 12 and 24 months, as shown in Figure 1). When indicated, additional CT scans or ultrasound investigations were carried out. During PCP visits, cardiovascular risk management (CVRM) was carried out.

**Figure 2:**
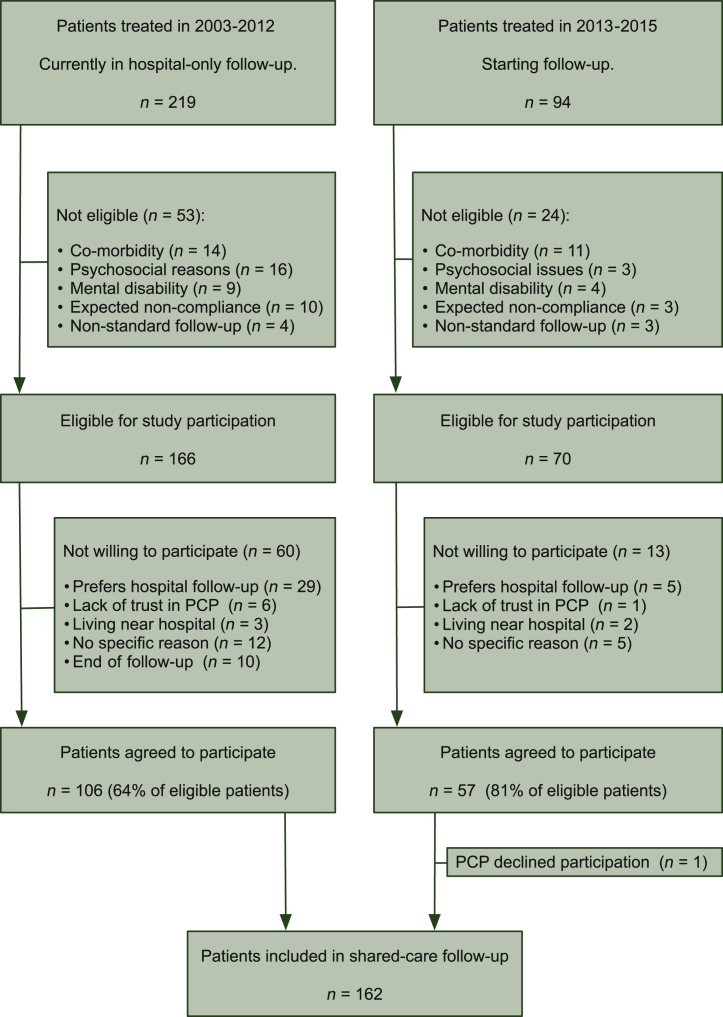
**Cohort diagram of the study population.** PCP, primary care physician.

To evaluate the safety of the shared-care survivorship program, a stopping rule was formulated regarding the rate of failures in relapse detection. Patients with metastatic testicular cancer who started with platinum-based chemotherapy after 1 January 2003 were eligible for the shared-care program (Figure 2). Inclusion criteria were complete remission after completion of chemotherapy followed by surgery in case of residual disease and ≥18 years of age. Exclusion criteria were significant co-morbidity, psychosocial issues, mental disability and expected non-compliance non-standard follow-up. The medical ethics committee of the University Medical Center Groningen approved this study. All participants gave written informed consent.

### Primary outcome

The primary outcome, as indicator of safety of the shared-care program, was failed detection and/or failed response in case of relapsed disease. This was tested by the use of a stopping rule. This stopping rule acted as a statistical instrument for continuous monitoring of failures in relapse detection (Supplementary Appendix Box A1, available at https://doi.org/10.1016/j.esmoop.2022.100488); this concept was previously used successfully in a study by Van der Zee et al.^10^ If during a follow-up visit abnormal findings indicating potential relapsed disease were ascertained, appropriate action should be initiated within 2 weeks; otherwise, it would be deemed as a failure.

The rate of failed relapse detection during the standard oncologist-only follow-up was estimated to be 5%. An increase in failed relapse detection of 5% was considered to be the maximum acceptable increase. Inferiority of the failure rate to 0.05 (H0: *P* = 0.05; H1: *P* = 0.10) was tested in a fully sequential design. A concomitant test of superiority to 0.10 (H0: *P* = 0.10; H1: *P* = 0.05) was used as a test for futility. For both tests, a constant value of α was used, cumulating to 0.05 with 80% power. The medical ethics committee of our hospital would be informed on the activation of the stopping rule and would be requested to judge the study based on the results of this analysis, regarding the following: closure, or continuation with an amended protocol, or continuation with an unchanged protocol.

Our power calculation was based on the assumption that failed detection becomes evident within 2 years of shared-care follow-up, including at least two primary care visits. To exclude an increase in failure rate of 5%, 225 patients with 2 years of follow-up would be needed to reach a one-sided level of significance of 5% with 80% power. With final analysis 2 years after the last entry and an accrual within 18 months of the study, a non-evaluable rate of 10% brought the needed number of patients to 245. The number of patients needed to reach significance would be lower if the number of failures remained low.

### Secondary outcomes

Secondary outcomes were the proportion of carried out CVRM assessments in primary care and psychosocial status.

During CVRM assessment, blood pressure, weight, body mass index and waist circumference were measured. Furthermore, laboratory measurements consisted of lipid levels, glucose and hemoglobin A1C and were carried out before the visit. The SCORE model was used to estimate 10-year risk for cardiovascular mortality and morbidity.^11^^,^^12^ To account for the increased cardiovascular risk of testicular cancer survivors, 15 years were added up to the actual age of patients. Based on their national CVRM guideline, PCPs provided lifestyle advices or prescribed medication.^13^^,^^14^

Psychosocial status was assessed with the Hospital Anxiety and Depression Scale questionnaire, subscale anxiety (HADS-A), and with the RAND-36 questionnaire, a multidimensional measurement of health.^15^^,^^16^ Both questionnaires have been extensively validated and carried out well in a wide range of populations.^17^

To evaluate the shared-care program, questionnaires for patients and PCPs were constructed to measure experiences with the shared-care program. The questionnaires were completed after the second primary care visit.

### Study procedures

If both the patient and his PCP agreed to participate, a personal survivorship care plan (SCP) was prepared by the study team. The SCP was generated with diagnosis and treatment data and based on national guidelines.^13^^,^^14^^,^^18^ The SCP defined the time points in follow-up for assessment of remission status and CVRM. The SCP also determined which visits would take place in primary care instead of the oncology center. The SCP was available both as a paper document and a mobile application.^19^ The PCP received the SCP and additional study instructions regarding CVRM and the same physical examination that was normally carried out by the oncologist. After each primary care visit, all information had to be sent back to the coordinating center within 2 days.

Shortly before each primary care visit, a blood sample was collected at the local practice of the PCP or a nearby hospital and sent to the coordinating center for central tumor marker evaluation [α-fetoprotein, β-human chorionic gonadotropin, lactate dehydrogenase (LDH)]. This process was monitored by a central data manager. A telephone appointment between a study physician and the patient was arranged shortly after the PCP visit to communicate the results of tumor marker measurement, to receive feedback and to plan the next visit at the coordinating center.

### Statistical analysis

Continuous variables were described with median and range and categorical variables were described with counts and proportions to summarize the study population and the characteristics of the primary care visits. All tests were two-sided and conducted at the 0.05 significance level. Statistical analyses were carried out by using IBM SPSS statistics 23 (IBM Corp., Armonk, NY).

## Results

### Study cohort

Between October 2012 and January 2017, 162 patients were enrolled in the shared-care program (Figure 2) and 150 PCPs participated (some PCPs had multiple participating patients in their practice). Overall, 69% of all eligible patients agreed to participate in shared-care follow-up. Of patients with <1 year of follow-up, 81% agreed to participate, compared to 64% of those with >1 year of follow-up. The most important reason to refuse participation was preference of hospital-only follow-up (14%); other reasons were lack of trust in their PCPs (3%) or a residence near the hospital (2%). One PCP declined to participate (99% participation, *n* = 150). Two other PCPs revoked their participation shortly after they agreed to participate in the study. Patients lived within a radius of 140 km to the University Medical Center Groningen (Supplementary Figure S1, available at https://doi.org/10.1016/j.esmoop.2022.100488). Diagnosis and treatment characteristics of the study participants are summarized in Supplementary Table S1, available at https://doi.org/10.1016/j.esmoop.2022.100488. During the trial, 30 patients stopped their participation in the shared-care program, due to a number of reasons shown in Supplementary Figure S2, available at https://doi.org/10.1016/j.esmoop.2022.100488.

### Safety of shared-care follow-up

One hundred and thirteen patients completed 2 years of shared-care follow-up with at least two primary care visits. In total, 364 visits in primary care took place (Supplementary Figure S2, available at https://doi.org/10.1016/j.esmoop.2022.100488). PCPs spent a median of 20 min (range 10-60 min) on the follow-up visit.

Six relapse cases were detected in the shared-care follow-up program, of which two occurred before the safety boundary was crossed (description of cases in Supplementary Table S2, available at https://doi.org/10.1016/j.esmoop.2022.100488). No failures in response to relapsed disease were observed. All relapses were detected during oncology visits with tumor marker assessments or CT scan imaging. In one relapse case, the preceding visit was a primary care visit. During this preceding visit, there were no indications of a tumor relapse. Another patient was referred by the PCP to a urologist, based on abnormal findings during physical examination. Subsequent ultrasonic imaging of the remaining testicle revealed no abnormalities.

**Figure 3:**
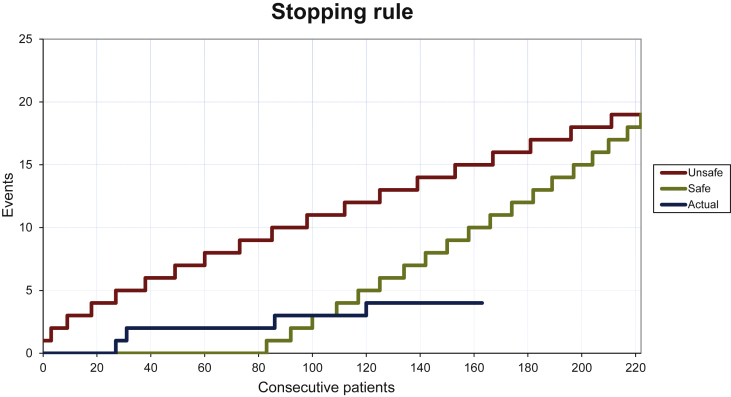
**Stopping rule for patients participating in the shared-care follow-up program.** The red line indicates inferiority to adequate relapse detection rate of 0.10; the green line indicates superiority to 0.05. The green (‘safe’) boundary was passed by the blue events line after enough patients with shared-care follow-up had completed 2 years of follow-up without a failure in detection of relapsed disease. Patients were censored at the time of end of shared-care follow-up by causes other than relapsed disease.

Figure 3 shows the performance of the stopping rule. Four patients had a visit that was considered as a failure because of organizational issues. Three primary care visits were considered as failed visits, because the patients did not make an appointment for a primary care visit and the coordinating center was not able to contact the patient within 2 weeks. Although in these cases no relapsed disease was detected, the inability to assess tumor risk led to the conclusion to consider these visits as failed visits. In one primary care visit, a wrong tumor marker was measured and this was not detected by the monitoring case manager. Therefore, this visit was also considered as a failed visit.

Nonetheless, these four failures did not cause activation of the stopping rule. Eventually, the safety boundary was crossed and thereby, the shared-care follow-up can be considered safe (Figure 3).

### Cardiovascular risk management

Of the participating patients, 140 (86%) had at least one primary care visit in which CVRM was carried out (Table 1). Metabolic syndrome was present in 25% of the patients. In 5% of the patients, the estimated 10-year risk for cardiovascular mortality or morbidity was ≥20%. In all cases of increased cardiovascular risk, the response of the PCP was timely and in accordance with the CVRM guideline.^13^^,^^14^ Lifestyle recommendations were given frequently, but medication prescription was limited in number. In 40 of 140 (28%) individual visits, the PCP did not report a conclusion about the calculated cardiovascular risk category.

**Table 1:** Cardiovascular risk management during primary care visits (*n* = 140)

	Median	Range
Age at follow-up visit (years)	38.2	20.5-75.7
Follow-up duration (years)	3.3	0.6-11.5

Cardiovascular risk profile	*n*	%
Metabolic syndrome	27/105	25
SBP ≥ 140/DBP ≥ 90 mmHg	29/130	22
Antihypertensive medication	10	7.4
Statin medication	2	1.5
Antidiabetic medication	2	1.5
Current smoker	31	22

	Median	Range
Systolic blood pressure^a^	125	95-166
Diastolic blood pressure^a^	78	50-109
Waist circumference (cm)	92	67-128
HDL cholesterol (mmol/l)^a^	1.30	0.7-9.0
Triglycerides (mmol/l)^a^	1.30	0.48-6.0
Glucose (mmol/l)^a^	5.3	3.3-8.1

Estimated cardiovascular risk (SCORE)	*n*	%
0%-10%	76	54
10%-20%	18	13
≥20%	7	5
Not reported by PCP	40	28

Outcome CVRM at primary care visit	*n*	%
Advice: Stop smoking	27	20
Advice: Lose weight	40	29
Advice: More physical exercise	29	21
New medication prescribed	5	4

CVRM, cardiovascular risk management; DBP, diabolic blood pressure; HDL, high-density lipoprotein; PCP, primary care physician; SBP, systolic blood pressure.

a Patients using medication excluded.

### Psychosocial status

The psychosocial questionnaires were carried out at inclusion in the study program and after a median of 18 months (range 1-77 months) of shared-care follow-up. Symptoms of anxiety were experienced by 6.4% of the patients. In the longitudinal analysis, anxiety levels did not change during shared-care follow-up (mean HADS-A 3.2 versus 3.2, *P* = 0.16) (Supplementary Table S3, available at https://doi.org/10.1016/j.esmoop.2022.100488). The RAND-36 questionnaire scores on the subscales ‘physical functioning’ and ‘role limitation due to physical problems’ improved during follow-up (Supplementary Table S3, available at https://doi.org/10.1016/j.esmoop.2022.100488).

### Evaluation of shared-care follow-up

Data from the evaluation questionnaire are presented in Table 2. Regular contact with their PCP was appreciated by 89% of the respondents and 86% would recommend shared-care follow-up to other patients. Overall, most PCPs considered shared-care follow-up as suitable for testicular cancer as well as for other types of cancer.

**Table 2:** Evaluation questionnaires (preliminary data on 145 patients and 150 primary care physicians)

Patients	Yes	No
Do you appreciate this organized care with involvement of your primary care physician?	89%	11%
Would you like to increase the part of follow-up visits in primary care?	40%	60%
Would you like to transfer follow-up care completely to primary care?	21%	79%
Would you recommend shared-care follow-up to other patients?	86%	14%
Did you experience the logistic procedures as an obstacle for shared-care follow-up?	41%	59%

Primary care physicians				
Shared-care follow-up could be applied for:	Agree	Agree somewhat	Disagree somewhat	Disagree
Testicular cancer	61%	23%	3%	7%
Breast cancer	39%	25%	15%	14%
Colon cancer	34%	24%	21%	14%
Prostate cancer	41%	29%	10%	11%

The logistic procedures were experienced as an obstacle by 41% of the patients and 21 patients (13%) withdrew their consent and decided to stop with shared-care follow-up, mainly because shared-care appointments were logistically more complex (e.g. one visit for PCP and a separate visit for laboratory measurements) or they had more confidence in hospital-based, oncologist-only follow-up (Supplementary Figure S2, available at https://doi.org/10.1016/j.esmoop.2022.100488).

With regard to organization, we experienced some logistic hurdles, mainly related to laboratory logistics and data reporting. In 26 of the 364 primary care visits, LDH was increased, probably due to hemolysis during transport, and reassessed within 2 weeks. In five other cases, tumor marker assessments had to be repeated due to incomplete measurements or faulty blood sample handling procedures.

After the visits, it was frequently observed that PCPs did not return the completed study forms in time. After 81 primary care visits (22%) the reports were not returned within 21 days to the coordinating center, but telephone contact with patient and PCP did not indicate signs of relapse risk. On the other hand, after 73 primary care visits (20%), the study form was sent within 1 day.

## Discussion

The results of this study indicate that this shared-care follow-up program for testicular cancer patients is a safe alternative for hospital-only follow-up. No failures were observed in the detection of relapsed disease. CVRM in shared-care follow-up also appears to be feasible. Patients did not become more anxious when in shared-care follow-up, and quality of life was not affected. Both patients and PCPs consider shared-care follow-up as a valuable substitute to hospital-based oncologist-only follow-up.

Transferring part of the follow-up to the PCP did not result in safety issues in this shared-care program. All cases of relapse disease were adequately and timely detected. It should be noted that all cases were detected with tumor marker assessments or CT scan imaging. The PCP was not involved in these aspects of the follow-up. In one PCP visit, the wrong tumor marker was assessed, without being noticed by the central case manager. In any model of testicular cancer follow-up, monitoring of tumor markers will be an important determinant for safety. Other studies on the safety of follow-up in collaboration with the PCP are scarce. Grunfeld et al. concluded that primary care-led follow-up after breast cancer does not result in more recurrence-related serious clinical events.^9^

This study also demonstrates the functionality of a simple, well-designed SCP in testing a new model of survivorship care, as argued by Jacobsen et al. in a review of SCP studies.^20^ The benefit of an SCP lies in promoting health care delivery and the SCP enables patients to play a more active role in their own follow-up. It should be noticed that the safety of follow-up is affected by the compliance of patients with the SCP. Three patients did not make an appointment for a primary care visit in the scheduled month and it was subsequently difficult to get into contact with the patient. Although these patients had no indications of relapsed disease, this could potentially have resulted in a failure. A motivated self-responsible patient seems an important prerequisite in safe shared-care follow-up.

The participation of testicular cancer patients in this type of follow-up was moderate (64%) to high (81%), depending on the duration of the prior follow-up period in the hospital setting. The fact that PCPs were approached after their patients already agreed to participate is probably one reason for the high participation of PCPs. Many patients recruited for this study live relatively far from our cancer center. The reduction in traveling time is one of the clear benefits of shared-care follow-up. Patients who were in follow-up for >5 years were least likely to be willing to participate in shared-care follow-up. This could be viewed as a missed opportunity for long-term transition to primary care.

Cardiovascular risk assessment appears to be feasible during primary care visits. Most interventions were lifestyle recommendations, partly aimed at the large subgroup of smokers. It should be noted that the outcome of the cardiovascular risk assessment was not reported to the coordinating center in 28% of the visits. The evaluation questionnaire showed that both patients and PCPs have a positive attitude toward shared-care follow-up. However, a small proportion of patients dropped out of the study, mainly because of logistic issues or because they preferred the routine of hospital follow-up. With regard to organization, we also observed significant delays in receiving study information reports after the primary care visits.

In future, motivated patients, especially those who live far from their specialized cancer center, could be offered shared-care follow-up. Patients should be informed that this follow-up is safe regarding relapse detection. The oncologist should contact the PCP to explain shared-care follow-up and to ask whether he or she is motivated for participation. When in doubt about the motivation of the PCP or the patient, shared-care follow-up should not be instituted. In this case, an ‘add-on’ model in which the PCP only executes the CVRM is more feasible. The HADS-A questionnaire indicated that anxiety levels did not change during shared-care follow-up. The RAND-36 questionnaire revealed an increase in the subscales ‘physical functioning’ and ‘role limitation due to physical problems’. However, it is not certain whether this increase is clinically meaningful. The non-randomized design of the study limits further conclusions.

There are strong points and a few limitations to this study. Strengths of this study are the high level of PCP participation and the continuous safety monitoring of participating patients using the stopping rule. A limitation is the non-randomized design and the fact that patients are included at variable points of follow-up, ranging from start of follow-up right after treatment until several years after the treatment.

### Conclusions

Shared-care follow-up after cisplatin combination chemotherapy for testicular cancer is feasible for motivated patients with dedicated PCPs. For patients living further away from the cancer center, this type of follow-up can be preferable and will save considerable time and costs due to traveling. The program results in increased collaboration with the PCP without compromising the safety of follow-up. A simple, well-designed and monitored survivor care plan for both care providers and patients is an important prerequisite.

## Supplementary data

Supplementary Material

Figure A1
