# Supplementary material for: Shared-care survivorship program for testicular cancer patients: safe and feasible
Source: ESMO Open. 2022 May 13;7(3):100488. doi: 10.1016/j.esmoop.2022.100488 (PMC9271504; doi:10.1016/j.esmoop.2022.100488)
Supplement: Supplementary Material [file mmc1.docx]

**Appendix**

**Box A.1**

*Detailed Statistical Design*

To check the safety of our survivorship program. stopping rules were defined. The stopping rules regard the failure to timely detect a relapsed case of testicular cancer. The rules are based on sequential analysis. with α and β spending properties. The boundaries of the rules were derived from a higher incidence *I0* and a lower incidence *I1*. assuming binomial distribution. The upper unsafe boundary was approximated by the maximal number of events *UB* to be accepted after *n* consecutive patients: UB(n) = integer(n x I1 + (Z(alpha) + CTC) x SD(I1) + 0.5). The maximal number of patients needed is: N(ub) = (((Z(alpha) + CTC) x (I1 x (1 –I1))^0.5 + Z(beta) x (I0 x (1–I0))^0.5)/(I0 –I1))^2. In these equations. *CTC* is the so-called Christmas Tree Correction (J. Whitehead. The Design and Analysis of Sequential Clinical Trials [ed. 2]. Chichester. United Kingdom. Wiley. 1997). *Z(alpha)* and *Z(beta)* were constant and chosen in such a way that the cumulative probabilities to pass the boundary did meet the preset *Cum(alpha)* for *I1* and *Cum(beta)* for *I0*. In analogy. the approximation for the minimal number of events *LB* was: LB(n) = integer(n x I0 –(Z(alpha) + CTC) x SD(I0) + 0.5). The minimal number of patients needed was: N(lb) = (((Z(alpha) + CTC) x (I0 x (1 –I0))^0.5 + Z(beta) x (I1 x (1 –I1))^0.5)/(I0 –I1))^2. Because the boundaries were only rough approximations in case of low values of *I0* and *I1* and because the probability to pass one boundary was influenced by the probability to pass the other one. all probabilities were computed exactly. The rule for patients with signs of a disease relapsed. with I0 = 0.10. I1 = 0.05. Cum(alpha) = 0.05. and Cum(beta) = 0.80 is used.

The cumulative probabilities to pass the boundaries of the rules are used. Monitoring of the data. entered in a spreadsheet. is done on a weekly basis. A member of the study team. either a medical doctor or a data manager. enters events on a daily basis. The stopping rule would be activated when the upper (unsafe) boundary is passed after k patients. irrespective of the individual follow-up intervals of the k patients or when neither boundary is passed after the maximum number of patients have all completed their follow-up (figure 3).

Figure S.1. Regional distribution of patients participating in the shared-care program.

*(in separate PDF file)*

162 patients included in shared-care program

1st primary care visit

n=142

2nd primary care visit

n=112

3rd primary care visit

n=65

4rd primary care visit

n=37

5th primary care visit

n=8

**Reasons to get off the shared-care program along the way (n=30):**

Relapsed disease (n=6)

Moved to other region (n=3)

PCP withdraws participation (n=2)

No trust in PCP (n=9)

Non compliance (n=1)

Withdrawal of consent, without known reason (n=9)

Figure S.2. Shared-care follow-up: Visits in primary care (n = 364)

|  |  |  |  |  |  |
| --- | --- | --- | --- | --- | --- |
|  | **Table S.1.** Basic characteristics of the study cohort. | | | |  |
|  |  |  | n / median | % / range |  |
|  | Cohort size | | 162 | - |  |
|  | Age at start of chemotherapy (years) | | 33.6 | 17.1 - 73.8 |  |
|  | Age at inclusion in study (years) | | 36.8 | 19.6 - 74.4 |  |
|  | Follow-up duration at inclusion (years) | | 2.5 | 0.3 - 9.8 |  |
|  | Diagnosis | Non-seminoma | 104 | 64% |  |
|  |  | Seminoma | 58 | 36% |  |
|  | Disease stage | II | 118 | 73% |  |
|  | [Royal Marsden] | III | 14 | 9% |  |
|  |  | IV | 30 | 19% |  |
|  | IGCCCG-risk group | Good | 128 | 79% |  |
|  |  | Intermediate | 28 | 17% |  |
|  |  | Poor | 6 | 4% |  |
|  | Chemotherapy regimen | BEP | 139 | 86% |  |
|  |  | EP | 6 | 4% |  |
|  |  | COC | 9 | 6% |  |
|  |  | Other | 8 | 5% |  |
|  | Abbreviations: IGCCCG, International Germ Cell Cancer Collaborative Group, BEP, bleomycin, etoposide, cisplatin; COC, cyclophosphamide, vincristine, carboplatin. | | | |  |
|  |  |  |  |  |  |

|  |  |  | |  |  | |  | |  | |  | |  | |
| --- | --- | --- | --- | --- | --- | --- | --- | --- | --- | --- | --- | --- | --- | --- |
|  | **Table S.2.** Detection of relapsed disease during shared-care follow-up. | | | | | | | | | | | |  | |
|  |  |  | |  |  | |  | |  | |  | |  | |
|  | *Patients in shared-care follow-up with relapsed disease* | | | | | | | | | | | |  | |
|  | Case 1 | Tumor marker elevation at scheduled oncology visit. | | | | | | | | | | |  |  |
|  | Case 2 | Tumor marker elevation and accompanying back pain at scheduled oncology visit. | | | | | | | | | | |  |  |
|  | Case 3 | Detected on scheduled CT scan: slowly growing teratoma. | | | | | | | | | | |  |  |
|  | Case 4 | Tumor marker elevation at scheduled oncology visit. | | | | | | | | | | |  |  |
|  | Case 5 | Detected on scheduled CT scan: slowly growing teratoma. | | | | | | | | | | |  |  |
|  | Case 6 | Detected on scheduled CT scan: slowly growing teratoma. | | | | | | | | | | |  |  |
|  |  |  |  | | |  | |  | |  | |  |  | |
|  |  | | | | | | | | | | | |  | |

|  |  | | | | | | |  |
| --- | --- | --- | --- | --- | --- | --- | --- | --- |
|  | **Table S.3.** Psychosocial questionnaires: longitudinal assessment. | | | | | | |  |
|  |  | | | | | | |  |
|  | HADS – Anxiety (n = 113) | Inclusion  (t=0) | |  | Follow-up, median 18 months after inclusion  (range 1-77) | |  |  |
|  |  | Mean | S.D. |  | Mean | S.D. | P-value |  |
|  | Anxiety sum score | 3.6 | 2.7 |  | 3.2 | 2.7 | 0.16 |  |
|  |  |  |  |  |  |  |  |  |
|  |  | n | % |  | n | % |  |  |
|  | Sum score ≥ 8 (mild symptoms) | 6 | 5.3 |  | 4 | 3.5 |  |  |
|  | Sum score ≥ 11 (severe symptoms) | 3 | 2.7 |  | 5 | 4.4 |  |  |
|  |  |  |  |  |  |  |  |  |
|  | RAND-36 (n = 113) | Mean | S.D. |  | Mean | S.D. | P-value |  |
|  | Physical functioning | 89.6 | (15.1) |  | 92.6 | 12.5 | < 0.01 |  |
|  | Social functioning | 86.9 | (17.4) |  | 90.1 | 15.5 | 0.26 |  |
|  | Role limitation due to physical problems | 74.1 | (39.1) |  | 84.6 | 29.7 | < 0.01 |  |
|  | Role limitations due to emotional problems | 88.2 | (27.6) |  | 90.6 | 24.7 | 0.74 |  |
|  | Mental health | 82.6 | (11.4) |  | 81.6 | 11.5 | 0.28 |  |
|  | Vitality | 68.5 | (18.1) |  | 69.0 | 17.6 | 0.59 |  |
|  | Bodily pain | 91.2 | (15.7) |  | 91.4 | 15.6 | 0.88 |  |
|  | General health perceptions | 72.0 | (18.9) |  | 71.2 | 21.1 | 0.69 |  |
|  |  | | | | | | |  |
|  |  | | | | | | |  |
