# Supplementary figures and images for: Shared-care survivorship program for testicular cancer patients: safe and feasible
Source: ESMO Open. 2022 May 13;7(3):100488. doi: 10.1016/j.esmoop.2022.100488 (PMC9271504; doi:10.1016/j.esmoop.2022.100488)

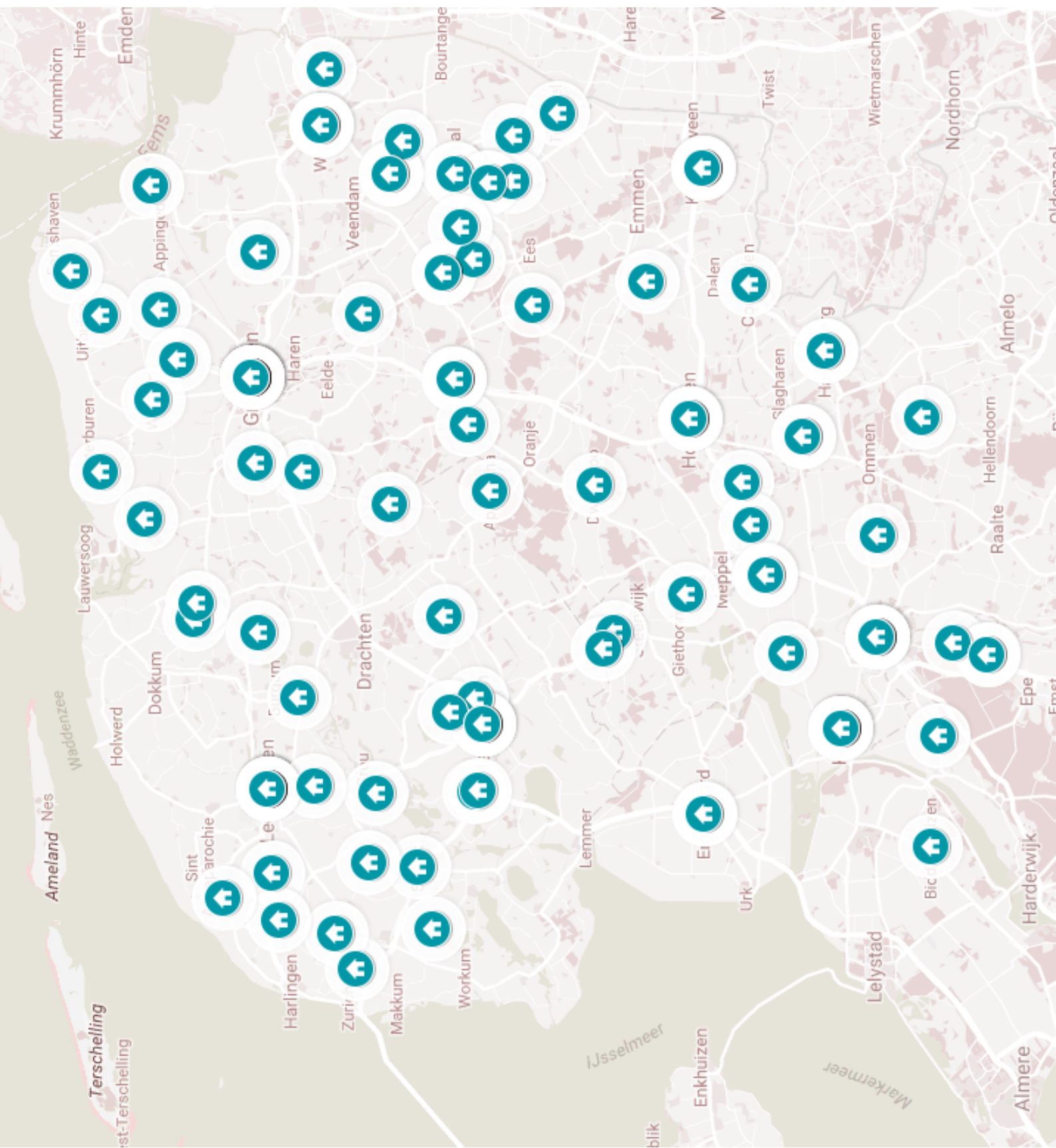

Supplement: Figure A1 [file mmc2.pdf]
